# Mortality associated with burn injury- a cross sectional study from Karachi, Pakistan

**DOI:** 10.1186/1756-0500-6-545

**Published:** 2013-12-19

**Authors:** Ehmer al Ibran, Farhat Hussain Mirza, Akhtar Amin Memon, Muhammad Zain Farooq, Maryum Hassan

**Affiliations:** 1 Civil Hospital Karachi, Dow University of Health Sciences, Karachi, Pakistan; 2 Department of Forensic Medicine, Dow Medical College, Dow University of Health Sciences, Karachi, Pakistan; 3 Dow Medical College, Dow University of Health Sciences, Karachi, Pakistan

**Keywords:** Burn Injury, Mortality

## Abstract

**Background:**

Burn injuries are a major cause of medico legal deaths in Pakistan. The present study was conducted with the aim to assess the mortality rate related to different types of burns injuries.

**Findings:**

This was an observational prospective cross sectional study conducted in Burns Ward of Civil Hospital, Karachi during a period of two years from January 1^st^ 2010 to December 31^st^, 2011. Data was collected over a questionnaire containing demographic variables as well as date of burn, date of the death (if patient expired), total body surface area involved, cause and manner of burn. The data was statistically analyzed by SPSS v. 16. A total of 1979 patients were admitted to the department during the study period. Out of them, 715 died, hence a mortality rate of 36.12%. Out of the 715 patients, 380 (53.1%) were males and 335 (46.9%) were females. Mortality was highest in age-group 16–30 years (n = 395, 55.2%). Majority of the deaths were accidental (n = 685, 95.8%). Fire burns was found to be the most common cause of death (n = 639, 89.3%). 35% (n = 252) of the patients who died had more than 60% of total body surface area involved in burns.

**Conclusion:**

Measures must be taken to inform the general population of the possible causes of these injuries, and to enable the people to be prepared to face any such circumstances.

## Introduction

Burn injuries pose a high burden of fatalities on the health systems worldwide. About 90% of burn incidents occur in low to middle income countries, areas that are deficient in the essential means to decrease the rate and intensity of such injuries [1].

In United States, the mortality rate in relation to burn injuries was 5.3%, with higher age groups and greater-% total body surface area (TBSA) burned associated with greater fatality rates [2].

The reported mortality rate due to burns in different Asian countries is variable. Sharma et al. reported a relatively low annual mortality rate of 0.6 per 100,000 in Kuwait [3]. Mashreky et al. reported a mortality rate of about 2.2 per 100,000 in Bangladesh in the year 2011 [4]. A study from Iran reported an annual fatality rate of 5.6 deaths per 100,000 [5]. In 2004 only, the death rate due to burns in Iraq was reportedly 12.3 per 100,000, much higher than rate worldwide [6]. Such high rate was on account of the war in Iraq that began in 2004. A study from India reported an even higher mortality rate of about 15.1 per 100,000 in year 2003 [7]. It further reported suicide commitment by females to be a major contributor to the death rate [7].

The objective of our current study is to determine the fatality rate of burn patients in Karachi, the largest city of Pakistan. Our study has been conducted on the base of available data at the only major burn centre of the city, where there are maximum reported burn cases than any other healthcare centre. We also aim to assess the effect of cause of burn cases, type of burns, and the total body surface area (TBSA) involved in related to mortality in burn patients.

## Findings

This cross sectional descriptive study was conducted at the Burns Ward of Civil Hospital, Karachi over a period over a period of two years from January 1^st^ 2010 to December 31^st^, 2011.

A total of 1979 patients were admitted at the burns centre during the study duration including 57% males (n = 1129) and 43% females (n = 850).

A preformed questionnaire was filled for each patient that included demographics like age, gender, type of burn, cause of burn, date of birth, date of death (if patient expired) and TBSA (total burnt surface area).

The data was entered and analyzed on SPSS version 16. Frequency and percentages were calculated for qualitative variables whereas means, standard deviations were calculated for quantitative variables. P-values were calculated to determine the significance of the data (using Pearson Chi Square Test). The study was approved by the Ethical Review Board of the Dow University of Health Sciences, Karachi, Pakistan.

## Results

Out of 1979 patients admitted to the department during the study period, 715 died with a mortality rate of 36.12%. Reported males (n = 380, 53.1%) were significantly higher than females 335 (46.9%). Incidence was reported to be higher in females (n = 853), being 39.4% of the admitted female. In males, the death rate was comparatively less, being 33.6% of the 1129 males admitted [Table 1].

**Table 1:** Demographics, cause of burn, type of burn and TBSA in relation to mortality rate

	**Patient prognosis**			
	**Alive**	**Dead**	**Total**	**P-Value**
**Frequency:**	1129	715	1979	
**Gender:**				
Male	749 (66.4%)	380 (33.6%)	1129 (100%)	0.008
Female	515 (60.6%)	335 (39.4%)	850 (100%)	
**Age groups:**				
≥ 15 years	236 (82.5%)	50 (17.5%)	286 (100%)	0.000
16–30 years	639 (61.8%)	395 (38.2%)	1034 (100%)	
31–45 years	262 (59.8%)	176 (40.2%)	438 (100%)	
46–60 years	90 (60%)	60 (40%)	150 (100%)	
61 years ≤	37 (52.1%)	34 (47.9%)	71 (100%)	
**Cause of burn:**				
Accidental	1241(64.4%)	678 (35.3%)	1919 (100%)	0.000
Homicidal	11 (45.8%)	13 (54.2%)	24 (100%)	
Suicidal	12 (33.3%)	24 (66.7%)	36 (100%)	
**Type of burn:**				
Fire burn	969 (60.3%)	639 (39.7%)	1608 (100%)	0.000
Electric burn	150 (81.5%)	34 (18.5%)	184 (100%)	
Chemical burn	41 (70.7%)	17 (29.3%)	58 (100%)	
Scaled burn	88 (81.5%)	20 (18.5%)	108 (100%)	
Flash burn	16 (76.2%)	5 (23.8%)	21 (100%)	
**TBSA:**				
≥ 10%	301 (86.7%)	46 (13.3%)	347 (100%)	0.000
11–20%	381 (86.8%)	58 (13.2%)	439 (100%)	
21–40%	408 (68.2%)	190 (31.8%)	598 (100%)	
41–60%	109 (39.2%)	169 (60.8%)	278 (100%)	
61% ≤	65 (20.5%)	252 (79.5%)	317 (100%)	

Mortality was higher in the age group between 16–30 years (n = 395, 55.2%). It was also significant in age group 61 years and above where out of n = 71 patients, 34 died (47.8%) as shown in Table 1. The youngest patient to die of the burn injury was a two year old male while oldest were two adults, one male and one female, each of 90 years respectively.

In the cases of accidental burns (n = 685) 95.8% of the patients died; whereas in the cases of suicide (n = 36), 24 died; giving a death rate of 66.67% [Table 1].

Fire burns was found to be the most common cause of death (n = 639, 89.3%). It was also the one with highest death rate of about 39.7% [Table 1].

Overall, majority (n = 252, 35%) of the patients who died had 61% or more the TBSA involved. Furthermore, out of the 317 patients with TBSA 61% or greater, 79.5% died. On applying linear regression analysis, strong correlation was found between burn area of the body (TBSA) and mortality i.e. 1.8% with 1% increase in TBSA [Table 1].

## Discussion

We report a mortality rate of about 36% due to burn injuries in Karachi, Pakistan during our study duration. Burn injuries are associated with 5% of mortality globally. About 500 billion US $ is the overall expense per annum related to burn injuries [8].

Globally, several studies have reported mortality rate due to burn injuries. Olaitan et al. reported a 20% mortality rate in Osogbo, Nigeria [9]. A study from Rotterdam, Netherlands reported a fatality rate of 6.9% [10]. Similarly, Aldemir et al. from Turkey reported a death rate of 6.3% [11].

A study from Wah, Pakistan reported 29.7% death rate in burn patients [12]. Muqim et al. from Peshawar, Pakistan reported 19% [13], while Chaudhry IA reported about 21% burn fatality rate from Rawalpindi, Pakistan [14]. Our study has reported by far, the highest mortality rate. Karachi, being the largest and most populated city in Pakistan, and being the economic hub of the country, such high rates are troublesome.

Males are more usually victims of burn injuries as is evident by our study. However, female tend to have a higher mortality rate. Mortality was also high in age group more than 60 years, where about 47% patients expired. This is consistent with another study by Chaudhry IA [14].

TBSA was a significant association with death of patients, where about 80% patients with TBSA greater than 60% died. It corroborates and even surpasses the rate reported earlier by O’Mara et al. where 70% of patients with TBSA greater than 60% died [15]. A study in Pakistan also reported 50% TBSA, above which all the cases involved died [12].

Majority of the deaths in our patients was due to accidental causes, fire burns being the most common type. We should provide awareness of proper first aid responses that may prevent such cases to aggravate. Also, fire extinguishers should be made available at more places and people should be provided with awareness of their use.

## Competing interests

The authors declare that they have no competing interests.

## Authors’ contributions

MFH gave the idea of the study. IEA and MAA designed the study. MAA, FZ, HM prepared the synopsis for IRB approval. MFH and IEA reviewed and edited the synopsis. FZ and HM collected the data and entered in on SPSS. MAA analyzed the data on SPSS. MAA wrote the initial draft of manuscript. All authors reviewed and edited the manuscript. All authors read and approved the final manuscript.

## References

[B1] Murray CJL Lopez AD The global burden of disease: a comprehensive assessment of mortality and disability from diseases, injuries, and risk factors in 1990 and projected to 2020 2006 Swizerland: World Health Organization

[B2] Latenser BA Miller SF Bessey PQ Browning SM Caruso DM Gomez M National burn repository 2006: a ten-year review J Burn Care Res 2007 6 5 635 658 10.1097/BCR.0B013E31814B25B1 17969244

[B3] Sharma PN Predicting factors influencing the fatal outcome of burns in Kuwait Burns 2005 6 2 188 192 10.1016/j.burns.2004.09.020 15683691

[B4] Mashkarey SR Rahman A Svanstrom L Khan TF Rahman F Burn mortality in Bangladesh: findings of national health and injury survey Injury 2011 6 5 507 510 10.1016/j.injury.2009.11.020 20031124

[B5] Maghsoudi H Pourzand A Azarmir G Etiology and outcome of burns in Tabriz, Iran. An analysis of 2963 cases Scand J Surg 2005 6 1 77 81 15865123 10.1177/145749690509400118

[B6] WHO Annual incidence (000s) for selected causes, in WHO Regions (a), estimates for 2004 [online] 2004 [cited 2009 June 20]; Available from http://www.who.int/healthinfo/global_burden_disease/estimates_regional/en/index.html

[B7] Batra AK Burn mortality: recent trends and sociocultural determinants in rural India Burns 2003 6 3 270 275 10.1016/S0305-4179(02)00306-6 12706621

[B8] Nordberg E Injuries as a public health problem in Sub-Saharan Africa: epidemiology and prospect for control East Afr Med J 2000 6 1 4 12862115

[B9] Olaitan PB Jiburum BC Analysis of burn mortality in a burns centre Ann Burns Fire Disasters 2006 6 2 59 62 21991024 PMC3188036

[B10] Bloemsma GC Dokter J Boxma H Oen IM Mortality and causes of death in a burns centre Burns 2008 6 8 1103 1107 10.1016/j.burns.2008.02.010 18538932

[B11] Aldemir M Kara IH Girgin S Guloglu C Factors affecting mortality and epidemiological data in patients hospitalized with burns in Diyarbakir, Turkey S Afr J Surg 2005 6 4 159 162 16440590

[B12] Khan N Malik MA Presentation of burn injuries and their management outcome J Pak Med Assoc 2006 6 394 397 17091750

[B13] Muqim R Zareen M Dilbag Hayat M Khan I Epidemiology and outcome of burns at Khyber teaching hospital Peshawar Pak J Med Sci 2007 6 420 424

[B14] Chaudhry IA Burns: frequency and mortality related to various age groups J Surg Pak 2009 6 2 67 71

[B15] O’Mara MS Caushaj P Goldfarb IW Slater H Treatment and mortality trends among massively burned patients Ann Burns Fire Disasters 2000 6 2 73

